# Comprehensive Evaluation System for Post-Metabolic Activity of Potential Thyroid-Disrupting Chemicals

**DOI:** 10.4014/jmb.2301.01036

**Published:** 2023-06-12

**Authors:** Yurim Jang, Ji Hyun Moon, Byung Kwan Jeon, Ho Jin Park, Hong Jin Lee, Do Yup Lee

**Affiliations:** 1 Interdisciplinary Program in Agricultural Genomics, Seoul National University, Seoul 08826, Republic of Korea; 2 Department of Agricultural Biotechnology, Seoul National University, Seoul 08826, Republic of Korea; 3 Department of Bio and Fermentation Convergence Technology, Kookmin University, Seoul 02707, Republic of Korea; 4 Department of Food Science and Biotechnology, Chung-Ang University, Anseong 17546, Republic of Korea; 5 Center for Food and Bioconvergence, and Research Institute of Agriculture and Life Sciences, CALS, Seoul National University, Seoul 08826, Republic of Korea

**Keywords:** Thyroid-disrupting chemical, biotransformant prediction, metabolomics, molecular networking, reporter gene assay

## Abstract

Endocrine-disrupting chemicals (EDCs) are compounds that disturb hormonal homeostasis by binding to receptors. EDCs are metabolized through hepatic enzymes, causing altered transcriptional activities of hormone receptors, and thus necessitating the exploration of the potential endocrine-disrupting activities of EDC-derived metabolites. Accordingly, we have developed an integrative workflow for evaluating the post-metabolic activity of potential hazardous compounds. The system facilitates the identification of metabolites that exert hormonal disruption through the integrative application of an MS/MS similarity network and predictive biotransformation based on known hepatic enzymatic reactions. As proof-of-concept, the transcriptional activities of 13 chemicals were evaluated by applying the in vitro metabolic module (S9 fraction). Identified among the tested chemicals were three thyroid hormone receptor (THR) agonistic compounds that showed increased transcriptional activities after phase I+II reactions (T3, 309.1 ± 17.3%; DITPA, 30.7 ± 1.8%; GC-1, 160.6 ± 8.6% to the corresponding parents). The metabolic profiles of these three compounds showed common biotransformation patterns, particularly in the phase II reactions (glucuronide conjugation, sulfation, GSH conjugation, and amino acid conjugation). Data-dependent exploration based on molecular network analysis of T3 profiles revealed that lipids and lipid-like molecules were the most enriched biotransformants. The subsequent subnetwork analysis proposed 14 additional features, including T4 in addition to 9 metabolized compounds that were annotated by prediction system based on possible hepatic enzymatic reaction. The other 10 THR agonistic negative compounds showed unique biotransformation patterns according to structural commonality, which corresponded to previous in vivo studies. Our evaluation system demonstrated highly predictive and accurate performance in determining the potential thyroid-disrupting activity of EDC-derived metabolites and for proposing novel biotransformants.

## Introduction

Endocrine-disrupting chemicals (EDCs) are exogenous compounds that bind to hormone receptors and disrupt the endocrine system [1, 2]. They are found in various products, including food packaging, pesticides, pharmaceuticals, personal care products, and flame retardants. EDCs adversely affect the ecosystem as well as human health and can cause reduced fertility, metabolic disorders, cancers, and abnormal development [3, 4].

Accordingly, the OECD, in its guidelines provided to assess the critical effects of EDCs, has proposed a conceptual framework to evaluate their endocrine activity in five stages with standardized testing for each stage. In particular, Level 2 of the framework targets mechanistic data on selected compounds, in which in vitro assays are performed using cell lines. Numerous high-throughput analyses have been conducted, but were to evaluating the endocrine-disrupting activities of androgen receptors (ARs) and estrogen receptors (ERs) [5, 6].

Thyroid hormones (THs) are crucial for normal brain development and a deficiency can result in a range of neurological dysfunction [7, 8]. Thyroid dysfunction may also be caused by thyroid hormone-disrupting chemicals (TDCs). Nonetheless, no test guidelines or standardized testing methods for TDCs have been properly established [9].

In our previous study, we proposed an evaluation platform for TDCs using levothyroxine (T4) as a model compound [10]. The platform consists of the S9 fraction for in vitro metabolic reaction, reporter gene assay, and tandem mass spectrometry for the transcriptional activity of TDC-derived biotransformants. We proved that the platform could successfully characterize the activity changes by the metabolism of potential TDCs, and further validated the system with bithionol.

Our current effort expedites the system’s robustness and reliability for comprehensively characterizing THR agonistic activity by potential TDCs. The pipeline introduces molecular network analysis that greatly facilitates the identification scope and accuracy for metabolic features that are attributed to potential THR activity. As proof-of-concept, a total of 13 chemicals were comprehensively evaluated and coupled to reporter gene assay, which resulted in post-metabolic alteration and was accompanied by dynamic changes in the parental compound and the potential metabolized compounds. With further investigation and mode-of-action analysis, the evaluation platform could be applied to a high-throughput screening system to minimize time-consuming and labor-intensive procedures (*e.g.*, animal study) [11, 12].

## Materials and Methods

### Chemicals and Standards

The following standard chemicals were donated by the Ministry of Food and Drug Safety (MFDS): Levothyroxine (T4), 3,3’,5-triiodo-L-thyronine (T3), 3,5-diiodothyropropionic acid (DITPA), sobetirome (GC-1), bisphenol A (BPA), 2,2',4,4'-tetrahydroxybenzophenone (BP-2), 17β-estradiol (E2), hispidulin, procymidone, rafoxanide, closantel, amiodarone hydrochloride (Amiodarone), BDE No. 28 (BDE28), and 4,4'-diiodobiphenyl (4,4’-DIDBP). The following chemicals were purchased from Sigma-Aldrich (USA): Dimethyl sulfoxide (DMSO), bovine serum albumin (BSA), glucose-6-phosphate (G-6-P), glucose-6-phosphate dehydrogenase (G-6-PD), magnesium chloride (MgCl_2_), L-glutathione reduced (GSH), uridine 5'-diphosphoglucuronic acid trisodium salt (UDPGA), adenosine 3'-phosphate 5'-phosphosulfate lithium salt hydrate (PAPS), and nicotinamide adenine dinucleotide phosphate (NADPH). Potassium phosphate buffer (PBS, 0.5M) was purchased from iNtRON Biotechnology (Korea). Rat (Sprague-Dawley) pooled liver S9 fraction was purchased from Corning, USA). LC-MS-grade formic acid, ammonium acetate, water, methanol (MeOH), and acetonitrile (ACN) were purchased from Thermo Fisher Scientific (USA).

### Cell Culture

The hTRE_HeLa cell line, obtained from the MFDS, was used to detect thyroid hormone receptor (THR) agonists through transcriptional activation analysis, utilizing a stably modified thyroid receptor-reactive human cervical cancer cell line along with one plasmid from a nano luciferase reporter [13, 14]. For cell line maintenance, minimum essential media (MEM, Gibco, USA) supplemented with 10% FBS, 1% penicillin/streptomycin (P/S), and 1 ml Hygromycin B (InvivoGen, USA) was used. The hTRE_Hela cell line was conditioned and treated in test medium containing Phenol red free of MEM (Gibco) supplemented with 10% charcoal-stripped FBS (CS-FBS), 1% P/S, and 1 mL Hygromycin B. hTRE_HeLa cells were maintained in 37°C and 5% CO_2_ incubator. hTRE_HeLa cells were seeded with 5 × 10^3^ cells per well in the 96-well plate for 24 h and treated with EDCs and S9 mixtures for 48 h.

### In Vitro Metabolic Reaction Using Rat Liver S9 Fraction

The S9 mixture for phase I metabolism was prepared with 0.1 mg/ml rat liver S9, 2 × 10^-3^ M NADPH, 3 × 10^-2^ M G-6-P, 5 × 10^-2^ M MgCl_2_, and 3 units/ml G-6-PD. For phase I+II metabolism, 2 × 10^-2^ M GSH, 5 × 10^-3^ M UDPGA, and 2 × 10^-5^ M PAPS were additionally included to the S9 mixture. The inactive S9 group did not include any cofactors except rat liver S9. The S9 mixtures were added to the aforementioned test medium to be diluted 10 times [15]. After 24 h of seeding, hTRE_HeLa cells were treated with the S9 mixtures pre-incubated for 6 h and EDCs with different concentrations.

### Luciferase Assay

In the hTRE_Hela THR transcriptional activity assay, 100 nM of T3 was used as a positive control (PC). The vehicle control (VC) included only 0.145% of DMSO containing 9 × 10^-12^ M 9-cis-Retinoic acid. The final volume for each well was 100 μl. The activity of T3 (100 nM) showed at least a 4.8-fold induction compared to the VC value. After treatment, 10 μl of treated medium was transferred to a new plate, to which 40 μl of test medium and 50 μl of Nano-Glo luciferase reagent (Promega, USA) were added. After a 10 min incubation, the luciferase activity was measured using a luminescence reader (Glomax Discover, Promega). For data analysis, relative transcriptional activity (RTA) of PC was set to 100%, and RTA of VC_control_ was set to 0%. The % to PC value of EDCs was calculated to (EDCs – VC_test_) / (PC – VC_control_). The VC_test_ was set to the VC for each test group. If the EDC was greater than or equal to 10% of PC, the EDC was determined to be a THR agonist-positive chemical. The data were described by the mean ± standard deviation.

### Sample Preparation for Metabolic Profiling

Samples for metabolic profiling were prepared identically to our previous study [10], except for the concentration of each TDC. The concentration was determined considering the MS detectability (see Table S1 for details). Final composition of the reaction mixture was as follows: 0.25% (w/v) of BSA, 20% (v/v) of PBS (0.5M, pH 7.4), 2 × 10^-4^ M of NADPH, 3 × 10^-3^ M of G-6-P, 5 × 10^-3^ M of MgCl_2_, 0.3 unit/ml of G-6-PD and 0.01 mg/ml of rat liver S9 fraction. For phase I+II reactions, 2 × 10^-3^ M GSH, 5 × 10^-4^ M UDPGA, and 2 × 10^-6^ M PAPS were added [15]. In the case of the negative control group, the S9 fraction was inactivated by organic solvent (ACN:MeOH, 1:1, v/v) at 5 times the volume of the S9 fraction. Ten microliters of TDCs were added to the reaction mixture (490 μl), which resulted in 500 μl of final working volume. The organic solvent mixture (1 ml of ACN:MeOH, 1:1, v/v) was added to 200 μl of sample to extract biotransformants. For deproteinization, the extract mixture was stored at −20°C for 1 h. The supernatant was isolated after centrifugation (16,100 ×*g*, 15 min, 4°C) and evaporated in a speed vacuum concentrator (SCANVAC, Korea). Dried residual was kept at −80°C in a freezer until further analysis.

### LC-MS Analysis

The dried residual was reconstituted with 200 μl of 50% ACN (v/v). The Ultimate-3000 UPLC system (Thermo Fisher Scientific) was used with a Hypersil Gold C-18 column (1.9 μm, 2.1 × 100 mm, Thermo Fisher Scientific) for chromatographical separation. Chromeleon 6.8 operation software (Dionex, USA) controlled the LC system. The mobile phase was composed of solvent A (DW) and solvent B (ACN) which included 0.1% formic acid (v/v) or 10 mM ammonium acetate. The gradient elution program was run using the following conditions: 0–2.0 min, 10% B; 2.0–20.0 min, 10%–95% B; 20.0–22.0 min, 95% B; 22.0–22.1 min, 95%–10% B; 22.1–25.0 min, 10% B. Flow rate was 300 μl/min. The mass spectrometric analysis was executed by Q-Exactive Plus Hybrid Quadrupole-Orbitrap Mass Spectrometer (Thermo Fisher Scientific), which was regulated by Q-Exactive Tune and Xcalibur 4.0 software. The ionization mode was set to positive or negative (see Table S1 for details). The spray voltage of the HESI-II probe was set to 3.8 kV for positive ionization and 3.5 kV for negative ionization. Ionized samples were fully scanned from 150 to 2,000 m/z, with resolution of 70,000 FWHM. The MS/MS spectra were obtained in a data-dependent manner with stepped normalized collision energy (NCE) of 30-40-50 eV and resolution of 17,500 FWHM [10].

### GC-MS Analysis

Samples for BDE28 and 4,4’-DIDBP were analyzed by GC-MS for estimation of metabolic rates since the compounds were not detected by LC-MS analysis. The dried residual was derivatized according to our previous study [16] with minor modification, in which the final volume of the derivatized sample was 25 μl. The derivatized sample was analyzed using an Agilent 7890B Gas Chromatograph (Agilent Technologies, USA) coupled to a Leco Pegasus HT time-of-flight mass spectrometer (LECO, USA) as previously described [17, 18].

### Data Processing

The LC-MS raw data was processed using Compound Discoverer 3.1 software (Thermo Fisher Scientific) [19-21]. The raw data were input to our workflow for reaction-based prediction and untargeted metabolic profiling, which was established in our previous study [10]. Chromatogram and spectral data were processed through the processing nodes (Select Spectra, Align Retention Times, and Detect Compounds), which were set to 5 ppm of MS1 tolerance, 10,000 counts of minimum peak intensity, and 3 of signal-to-noise ratio threshold. Known biochemical reaction-based feature annotation (expected compound module) was conducted according to the following computational workflow: Generate Expected Compounds, Find Expected Compounds, and FISh scoring nodes. All phase I and II transformation libraries were applied, and deiodination reaction was manually added to transformation libraries for iodine-containing compounds (*e.g.*, T3, DITPA, amiodarone, and 4,4’-DIDBP). Maximal number of reactions was set to 5 for total reactions and 1 for phase II reactions. Predictive compounds were found and grouped based on precursor mass with 5 ppm of mass tolerance and 0.1 min of RT tolerance. Fragment Ion Search (FISh) scoring was calculated with 10 ppm of mass tolerance and 3 of signal-to-noise ratio threshold only for compounds with MS2 spectral data. The resultant processed data and predicted data were merged in Merge Features node and searched in mZCloud library. The resultant data matrix was filtered by 105 of maximum peak area or 50 of FISh coverage score [22].

### Statistical Analysis

Statistical analysis was executed on all continuous variables obtained from LC-MS and GC-MS. To test statistical significance, the Mann-Whitney U-test was implemented in Multiple Array Viewer v.4.9.0 software (http://mev.tm4.org/)[23]. Multivariate statistical analysis was performed using SIMCA 15 (Sartorius, Göttingen, Germany). Volcano plots were created using GraphPad Prism v.7.04 (GraphPad Software Inc, USA). Bar plots were generated based on *LMSstat* version 1.0.11 package (https://github.com/CHKim5/LMSstat) in R version 4.0.4 and RStudio version 1.3.1073.

### Molecular Network Analysis

The molecular network was constructed based on the feature-based molecular networking (FBMN) workflow [24] on GNPS (https://gnps.ucsd.edu/ProteoSAFe/static/gnps-splash.jsp) [25, 26]. Mass features ([M+H]+adducts) were identified using the following parameters: MS1 tolerance = 0.01 Da, MS2 tolerance = 0.025 Da, Maximum charge = 2, Mass range = 100-1000, Minimum peak height = 10,000 counts. For MS2 deconvolution, a sigma window value of 0.5 was used, with an MS/MS abundance cut-off of 1,000. The features were aligned to pooled-sample quality controls with 0.1 minute retention time tolerance and 0.02 Da MS1 tolerance. For missing mass features, values were imputed with 10% of the minimum peak height over all samples [27].

Mass tolerance of precursor ion and fragment ion was set to 0.05 Da. Edges were connected only when cosine score was more than 0.5 and have more than 6 matched fragment ions. The maximum shift between precursors was set to 500 m/z and the maximum number of neighbors was 10 nodes. The constructed nodes were then annotated based on the GNPS spectral library. The MolNetEnhancer workflow was further applied to the network constructed by the FBMN platform. Chemical class of total network features was annotated by the ClassyFire module (https://jcheminf.biomedcentral.com/articles/10.1186/s13321-016-0174-y) in the MolNetEnhancer workflow [28-30]. Cytoscape 3.8.2 software was applied for visualization.

### Chemical Structure

Chemical structures of the biotransformants were predicted from the Compound Discoverer 3.1 software, *BioTransformer* version 3.0.0 [31] and *SyGMa* version 1.1.1 [32]. Chemical structures were described using ChemSketch software 12.01 (ACD/Labs, Toronto, Canada).

## Results and Discussion

### THR Agonistic Activities Are Significantly Altered by the Biotransformation of 4 Compounds – T4, T3, DITPA, and GC-1

**Fig. 1:**
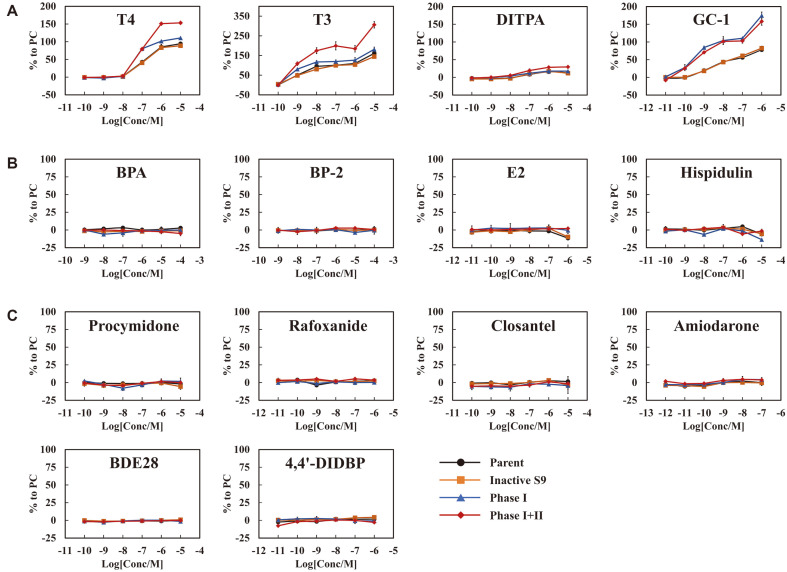
Thyroid receptor (THR) agonistic activities of EDC-derived metabolites in the hTRE_HeLa cells. Test groups include parent group (black), inactive S9 group (orange), Phase I group (blue), and Phase I+II group (red). The results are expressed as the means ± standard error (*n* = 3). (**A**) The activity profiles of THR agonistic positive compounds (T4, T3, DITPA, and GC-1). (**B**) The activity profiles of organic compounds that contain only carbon, hydrogen, and oxygen with THR agonistic negative activities (hispidulin, BPA, E2, and BP-2) (**C**) The activity profiles of halogen compounds that show THR agonistic negative activities (procymidone, closantel, amiodarone, rafoxanide, BDE28, and 4,4'-DIDBP).

THR agonistic activity was assayed on 14 compounds including T4 as cross-validation, which was reported in our previous study. Our previous study demonstrated that the S9 fraction and cofactors did not inhibit the transcriptional activity of T4 and its metabolites [10]. Likewise, all the compounds showed no significant difference in THR agonistic activities between parent compounds and products that underwent S9 reaction without cofactor (Fig. 1, Inactive S9 group, orange line). The equivalent activity indicates that the S9 fraction itself does not affect the transcriptional activity of EDCs.

All THR activity evaluation experiments were conducted at concentrations that did not show cytotoxicity. EDCs (any concentrations) showing activity greater than 10% of the activity of 100 nM T3 (an endogenous thyroid hormone) is considered a THR agonistic positive compound. Among 14 compounds tested, 4 compounds showed THR agonist- positive activities in a dose-dependent manner and the activity changed after the S9 reaction (Fig. 1A). The other 10 chemicals did not show any THR agonist-positive activities before and after the S9 reaction (Figs. 1B and 1C). Interestingly, the four compounds showed increased activities after S9 treatment, particularly following phase I+II group. T3 and DITPA, which have two aromatic rings and iodine residues similar to T4, showed similar activity patterns to T4 (Fig. S1). Compared to the activity of 100 nM T3, the greatest activity (309.1± 17.3%) was obtained from 10 uM of the T3-biotransformant, which underwent phase I+II. The transcriptional activity of DITPA was slightly increased up to 30.7 ± 1.8% by phase I+II. GC-1 has a similar structure to the other 3 compounds, but it has methyl and isopropyl groups instead of iodine residue (Fig. S1). GC-1 showed a significant increase in transcriptional activities compared to the parent group in both phase I and I+II (166.3 ± 12.0% in phase I group, 160.6 ± 8.6% in phase I+II group).

### T3, DITPA, and GC-1 Showed Significant Metabolic Conversion and Associated THR Agonistic Activities with Common Metabolic Reactions

The metabolic profiles were interrogated focusing on the compounds that showed differential activities after S9-phase I+II biotransformation. Metabolic rate of T3, DITPA, and GC-1 were decreased by 3%, 29%, and 15%, respectively after the reaction. DITPA and GC-1 were significantly decreased by liver metabolism, but T3, the metabolite of T4, showed no significant change. Untargeted metabolic profiling resulted in 79, 70, and 47 features, respectively, for T3, DITPA, and GC-1 in combination with semi-targeted annotation based on our workflow of predicting biotransformants (see experimental section for details).

**Fig. 2:**
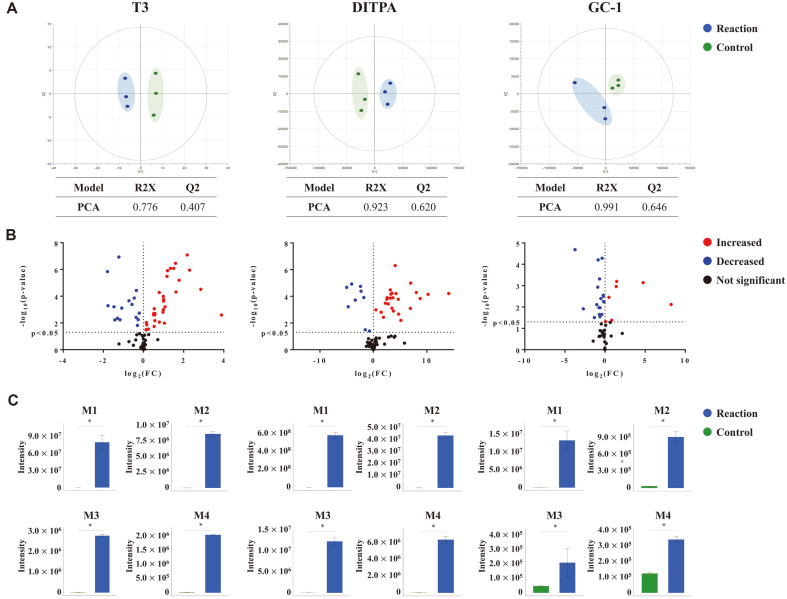
Metabolite profiles of the biotransformants with THR agonistic positive activity. The multivariate and univariate statistical analysis are applied to T3, DITPA, and GC-1, which show the significant changes of the activity. Variables are included, which are predictably annotated based on the potential biotransformants following the enzymatic reaction of Phase I and II. (**A**) The score plot of principal component analysis (PCA) model. Samples for T3, DITPA, and GC-1 (*n* = 3) are presented by PCA model. The R2Y and Q2 values indicate the fitness and predictive ability of the model, respectively. (**B**) The volcano plots of the biotransformants. The plot presents the fold change (*x*-axis) and statistical significance (*y*-axis) at the log-transformed scale. Red and blue nodes indicated significantly increased and decreased metabolites, respectively. Metabolites showing no significant changes are presented by black nodes. (**C**) Key biotransformants that undergo the most dramatic alteration. Error bar presents standard error of mean (SEM) and asterisk (*) indicates statistical significance (*p*-value <0.05, Mann Whitney U-test).

First, PCA score plots demonstrated distinctive patterns of EDC-derived biotransformants (Fig. 2A). The score plots of three compounds showed clear discrimination and excellent model power between the reaction group and the control group (R2Y = 0.776, Q2 = 0.407 for T3, R2Y = 0.923, Q2 = 0.62 for DITPA, and R2Y = 0.991, Q2 = 0.646 for GC-1).

T3 showed 33 biotransformants that were significantly increased while 20 metabolized compounds were downregulated after the phase I+II reaction (Fig. 2B, left panel). The glucuronide conjugation, the most common biotransformant [33], showed the highest increase after the phase I+II reaction of T3 (Fig. 2C, left panel). Similarly, the most dramatic increases were confirmed for the biotransformants, which underwent oxidation-glucuronide conjugation, desaturation-oxidation-taurine conjugation, and sulfation (Fig. 2C, left panel). For DITPA, 31 compounds were significantly increased and 9 compounds were significantly decreased (Fig. 2B, middle panel). The most increased biotransformants were glucuronide conjugation, oxidation-glucuronide conjugation, deiodination-deiodination-sulfation, and deiodination-glucuronide conjugation in descending order (Fig. 2C, middle panel). For GC-1, 10 and 21 biotransformants were significantly up- or downregulated, respectively, after the phase I+II reaction (Fig. 2B, right panel). Similar to T3 and DITPA, the highest fold increases were confirmed for the biotransformants such as glucuronide conjugation. Others were glucuronide conjugation-decarboxylation, oxidation-oxidation, and GSH conjugation (Fig. 2C, right panel).

Note that the major biotransformants of the compounds (T3, DITPA, and GC-1) were similar to those of T4 (glucuronide conjugation, sulfation, GSH conjugation, and amino acid conjugation) [10, 34, 35]. The deionized forms were also confirmed as the primarily metabolized compounds for T3 and DITPA. Other biotransformants known to be produced in vivo were also found in our data, including T3-glucuronide, T3-sulfate, T2-glucuronide, T2-sulfate [34, 36]; 3,3'-diiodothyropropionic acid (DITPA) sulfate, and 3'-monothyropropionic acid sulfate [37](Tables S2-S4). The results suggest that the S9 fraction-based biotransformation simulation at the in vitro level was as efficient as in vivo liver metabolism. Similar biotransformation patterns among the THR agonistic compounds (T3, DITPA, and GC-1) may be related to the increased levels of the agonistic activity by phase I+II reaction.

### Molecular Network Analysis Coupled to Targeted Predictive Annotation Empowers the Annotation of Biotransformants

Metabolic profiles were further interrogated to extend the scope of biotransformant annotation and to putatively link the features to the changes in the agonistic activity. Global natural products social molecular networking (GNPS) was applied to identify a broad range of unknown metabolic features based on spectral similarities. The molecular networking provides comprehensive access to the annotation of unknown compounds, which are experimentally generated, based on a vast library consisting of tandem mass spectrometry (MS/MS fragmentation pattern) [38, 39]. In addition, MolNetEnhancer was used to extremely expand the annotation range of the GNPS network. It is an effective tool that shows the overall chemical class changes of molecules, including unknown compounds. Common metabolic studies that rely on libraries do not consider the effects of unknown compounds, whereas molecular networking provides a wide range of understanding about unknown features.

**Fig. 3:**
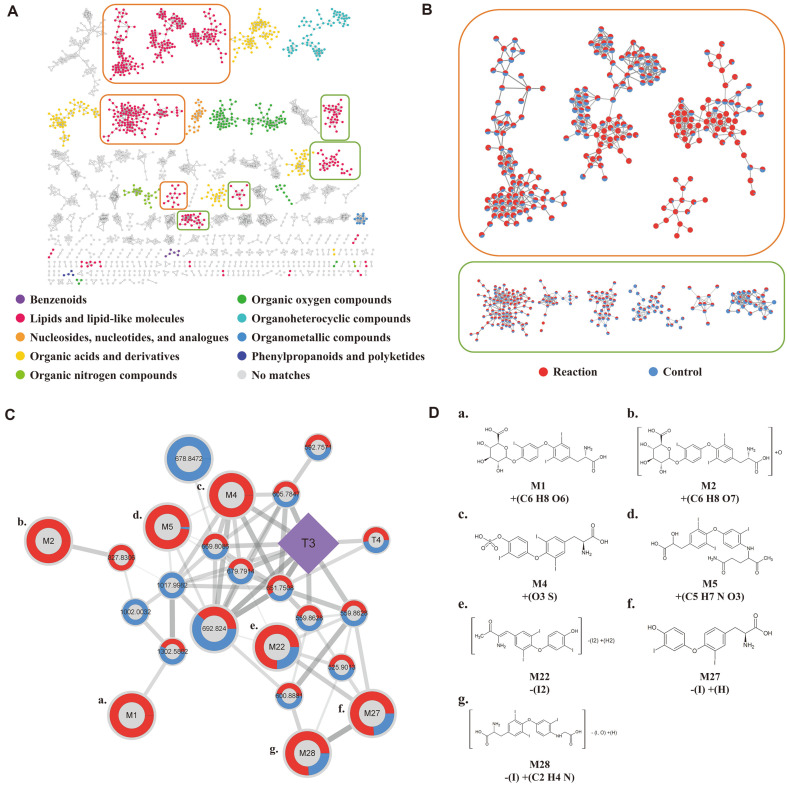
Enhanced annotation and quantitative evaluation of the untargeted profiles T3-biotransformants based on molecular network analysis. Molecular network is reconstructed, including all metabolic features that pertain correlation matrix based on mass spectral similarity by the Global Natural Product Social Molecular Networking (GNPS). (**A**) Molecular network of metabolic features from T3-biotransformation. Each node is a molecular feature with MS/MS. The node color indicates different chemical superclass. Grey-colored node is not assigned to known superclass. (**B**) The most distinctive alteration in the clusters of lipids and lipid-like molecules. Clusters in orange box are enriched by metabolic features that are significantly increased after Phase I and II reactions. Clusters in green box consist of metabolic features presenting marginal changes (increases or decreases). The nodes are presented as pie charts that show relative abundances between the reaction group (red) and the control group (blue). (**C**) Enlarged view of T3-containing subnetwork. The parent compound (T3) is indicated by a purple diamond. Pie charts on nodes visualize the relative peak intensities: the reaction group (red) and the control group (blue). For visual clarity, metabolic features with statistical significance are presented with bigger node size (*p* < 0.05, Mann-Whitney U-test). Edge width and transparency represents the cosine value (0.5≤Cosine value≤1.0). (**D**) Chemical structures of biotransformants identified in the T3-comprising cluster. Seven metabolic features are selectively presented, which are identified by both the molecular networking analysis and targeted annotation (expected compound module).

The GNPS network analysis resulted in 42 clusters (> 10 features) with 1,482 features out of 20,271 detected signals. The data were re-processed by the MolNetEnhancer module to acquire an overview of the metabolic features of chemical classes. Clusters annotated in the GNPS library were automatically classified into hierarchical chemical classes. The representative clusters were lipids, organic acids, and organic oxygen compounds. Particularly, lipids and lipid-like molecules were highly enriched (Fig. 3A, pink nodes). Among them were fatty acids or fatty acid derivatives that were identified as prevalent upregulation in the subclass (Fig. 3B, upper panel). Other glycerophospholipids and steroids-steroid derivatives showed marginal changes.

The cluster including T3 consisted of 23 nodes, in which 9 nodes were matched to the compounds annotated by the feature annotation by precursor m/z difference (Fig. 3C). The annotation of the metabolic features was curated by manual inspection of tandem mass spectra (Fig. 3D, Table S2). Each MS/MS fragment of the node was compared with the spectral pattern of biotransformants annotated from our reaction-based workflow. We annotated the final chemical structures by comprehensively considering the predicted reactions through our workflow and the predicted reaction positions through the various tools (Biotransformer and Sygma) [31, 32].

Note that the molecular network identified T4. We observed increased levels of T4 after biotransformation. Generally, T4 is converted to T3, T2, and T1 by consecutive deiodination reaction. Nonetheless, our data has shown the T4 generation from T3, which may indicate a potential “reverse reaction.” According to other research, iodination reaction can be catalyzed by peroxidase [40, 41]. This result showed that novel biotransformants that can be generated by unknown reaction are also predictable by our integrated workflow. Accordingly, the strategy combined with our targeted annotation (expected compound module) and the molecular networking demonstrated the possibility of comprehensive extension of annotation for novel biotransformants.

### Biotransformation Pattern Is Determined by Structural Specificity.

We speculated the profiles of 10 chemicals that did not show THR agonistic activity. The specific patterns of the resultant biotransformants were categorized according to the structural characteristic of the parent compounds (Table 1).

Organic compounds, only consisting of carbon, hydrogen, and oxygen (BPA, BP-2, E2, hispidulin) underwent the phase II reaction primarily. The most enriched biotransformant was glucuronide conjugation among the 4 compounds. Sulfation and GSH conjugation were also dominant reactions in the compounds. Previous studies have shown that the major metabolites of BPA, BP-2, and E2 are glucuronide and sulfate form in animal model and human study, which was consistent with our results [42-44]. In addition, significant amounts of the E2-conjugation forms were produced following the oxidation step, including oxidation-sulfation and oxidation-glucuronide conjugation, respectively. Hispidulin was biotransformed and annotated to 63 putative compounds. Although hepatic metabolism of hispidulin has not been previously studied, our results demonstrated that the major biotransformation was glucuronide conjugation, sulfation, and demethylation. The biotransformants agree with the forms of metabolized compounds that are produced from natural flavonoids (*e.g.*, apigenin) [45-47].

Halogen compounds with chlorine, bromine, and iodine (procymidone, rafoxanide, closantel, amiodarone, BDE28, 4,4’-DIDBP) showed relatively higher metabolic rates and higher numbers of biotransformants than the organic compounds described above (except for DIDBP and BDE28). The halogen compounds underwent a larger number of reactions than other compounds, and dehalogenation was the common reaction among them. In addition, the metabolized compounds showed structure-specific biotransformation patterns (procymidone-succinimide ring open, rafoxanide and closantel-amide hydrolysis, and amiodarone-deethylation) [48-50].

## Conclusion

In the current report, we extended and validated the platform for evaluating the post-metabolic effect of EDCs on thyroid-disrupting activities. The metabolic profiles of 13 chemicals showed unique biotransformation patterns according to the chemical structures. The metabolic profiles coupled with the transcriptional activities proposed the potential influence of TDC-derived metabolites based on our integrated platform in which an in silico feature annotation system and an in vitro reporter gene assay were systematically assembled.

The S9 fraction is a liver metabolic enzyme complex that decomposes external compounds and produces metabolites through complex biochemical pathways. Note that significant alteration of broad range of metabolites, which did not pertain to the EDC metabolism were identified based on molecular networking approach (*e.g.*, fatty acids). How the fatty acids underwent significant alteration by the EDC treatment remains elusive, but would be worthy of future study (*e.g.*, differential regulation of enzymes associated with fatty acid metabolism).

In addition, we extended the annotation coverage from target-oriented compounds to untargeted metabolites by pipelining the reaction-based prediction and spectral similarity-based molecular networking. Our results demonstrated the extensibility of annotating novel biotransformants that underwent a range of hepatic reaction including unexpected ones (*e.g.*, reverse reaction of T3). Future study should consider exploration into the detailed mechanisms of interactions between metabolites and receptor-binding abilities. Nonetheless, our systematic evaluation platform uncovers the influence of metabolites on endocrine-disrupting activities, and can be applied to toxicity evaluation for numerous exogenous compounds.

## Supplemental Materials

Supplementary data for this paper are available on-line only at http://jmb.or.kr.

## Figures and Tables

**Table 1:** Summary of biotransformants of compounds showing THR agonistic negative activity.

	Compound	Metabollic rate	Composition	MS1		MS2		Expected Compounds		
				Total	*p*<0.05	Total	*p*<0.05	Composition change	Transformation	FC
Organic compound	BPA	52%	C, H, O	7	6	5	5	+(C6 H8 O6)	Glucuronide Conjugation	8650.3
								+(C10 H15 N3 O6 S)	GSH Conjugation 1	130.3
								+(C H6 O2)	Hydration, Oxidation, Reduction, Methylation	2.8
	BP-2	11%	C, H, O	24	14	20	11	+(C6 H8 O6)	Glucuronide Conjugation	1466.1
								+(C6 H18 N4)	Hydration, Nitro Reduction, Reduction, Arginine Conjugation_B	102.2
								+(C6 H18 N4)	Hydration, Nitro Reduction, Reduction, Arginine Conjugation_A	83.7
								+(O3 S)	Sulfation	50.7
	E2	44%	C, H, O	26	13	17	10	+(C6 H8 O6)	Glucuronide Conjugation_B	593.8
								+(C6 H8 O6)	Glucuronide Conjugation_A	181.4
								+(O4 S)	Oxidation, Sulfation	63.0
								+(C6 H8 O7)	Oxidation, Glucuronide Conjugation	27.7
	Hispidulin	*-4%	C, H, O	63	34	26	16	+(C6 H8 O6)	Glucuronide Conjugation	1475.7
								+(O3 S)	Sulfation	86.3
								-(C) +(H2 O3 S)	Hydration, Nitro Reduction, Oxidation, Sulfation, Demethylation	31.5
								+(C5 H6 O5)	Desaturation, Glucoside Conjugation, Demethylation	28.4
Halogen compound	Procymidone	55%	C, H, O, N, Cl	98	58	28	22	-(H2 Cl_2_) +(O5 S)	Desaturation, Desaturation, Oxidative Dechlorination, Oxidative Dechlorination, Sulfation	1439.6
								-(C) +(H3 N3 O3)	Desaturation, Oxidation, Oxidation, Arginine Conjugation, Ring Open	344.6
								+(H2 O)	Ring Open	142.6
								+(H2 O6 S)	Oxidation, Oxidation, Sulfation, Ring Open	103.3
	Rafoxanide	51%	C, H, O, N, Cl, I	231	67	60	30	-(Cl I) +(C16 H34 O3)	Hydration, Oxidative Dechlorination, Deiodination, Palmitoyl Conjugation	52.6
								-(Cl I) +(C16 H32 O2)	Oxidative Dechlorination, Deiodination, Palmitoyl Conjugation_A	39.5
								-(Cl I) +(C16 H32 O2)	Oxidative Dechlorination, Deiodination, Palmitoyl Conjugation_B	16.7
								-(Cl_2_ I2) +(C3 H17 N3 O3 S)	Nitro Reduction, Oxidative Dechlorination, GSH Conjugation (on Chlorine), Hydrolysis	13.3
	Closantel	49%	C, H, O, N, Cl, I	181	54	40	23	-(C2 Cl_2_ I2) +(H6 N2)	Dehydration, Reduction, Methylation, -(C3 Cl_2_ I2) + (H4 N2 O2)	81.9
								-(Cl I) +(C5 H8 N2 O2)	Dehydration, Oxidative Dechlorination, Deiodination, Glutamine Conjugation	6.4
								-(I) +(C6 H9 O6)	Deiodination, Glutamine Conjugation	6.2
								-(Cl_2_ I) +(C6 H17 N4 O2)	Hydration, Reductive Dechlorination, Reductive Dechlorination, Deiodination, Arginine Conjugation	6.0
	Amiodarone	70%	C, H, O, N, I	91	52	28	18	+(O)	Oxidation	16.8
								-(C2 I N O3)	Nitro Reduction, Nitro Reduction, Oxidative Deamination to Alcohol, Deiodination, Deethylation	15.9
								+(C4 H3 N3 O3)	Desaturation, Oxidation, Oxidative Deamination to Ketone, Arginine Conjugation, Deethylation	11.6
								-(N) +(C14 H27 O)	Hydration, Nitro Reduction, Oxidative Deamination to Ketone, Palmitoyl Conjugation, Deethylation	8.4
	BDE28	99%	C, H, O, Br	44	14	11	3	-(H4) +(O3 S)	Desaturation, Desaturation, Sulfation	10.8
								+(C2 H4 O5)	Hydration, Oxidation, Oxidation, Oxidation, Acetylation	3.3
								-(Br) +(C2 H2 N O2 S)	Dehydration, Desaturation, Oxidative Debromination, Taurine Conjugation	3.2
								-(H Br) +(O2)	Desaturation, Oxidation, Oxidative Debromination	2.5
	4,4’-DIDBP	82%	C, H, I	6	2	1	0	+(C18 H32 O3)	Desaturation, Oxidation, Oxidation, Stearyl Conjugation	1.1

Fold change (FC) is calculated by mean area of the reaction group over the control group. Top 4 compounds with significant increases are presented in descending order (*p*<0.05, Mann-Whitney U-test). Composition change is predicted by precursor m/z difference in our targeted annotation (Expected compound module).

Transformation is predicted by the Expected compound module and additional manual curation.
