# Supplementary material for: Comprehensive Evaluation System for Post-Metabolic Activity of Potential Thyroid-Disrupting Chemicals
Source: J Microbiol Biotechnol. 2023 Jun 12;33(10):1351–60. doi: 10.4014/jmb.2301.01036 (PMC10619556; doi:10.4014/jmb.2301.01036)
Supplement: Supplementary file 1 [file jmb-33-10-1351-supple.pdf]

J. Microbiol. Biotechnol.
https://doi.org/10.4014/jmb.2301.01036

Supplementary Figures

Fig. S1. Chemical structure of tested TDCs

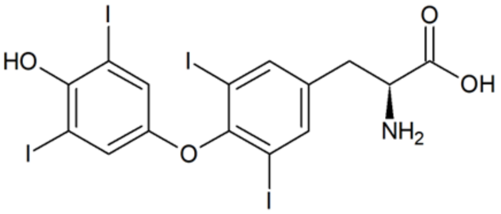

T4
(Levothyroxine)

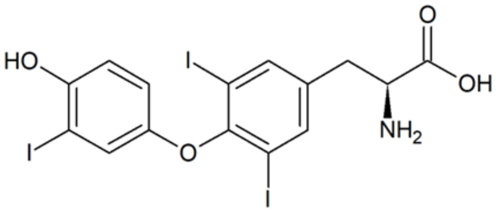

T3
(3, 3’, 5’-triiodothyronine)

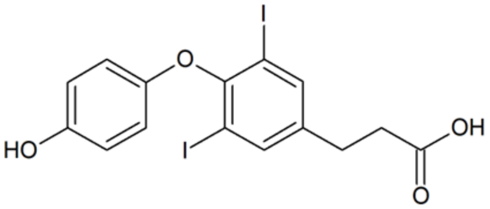

DITPA
(Diiodothyropropioninc acid)

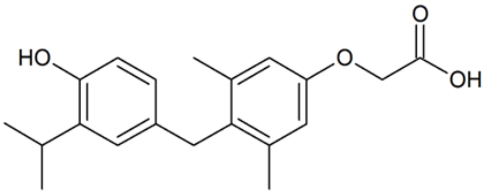

GC-1
(Sobetirome)

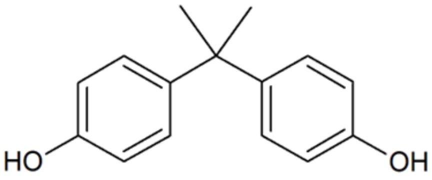

Bisphenol A

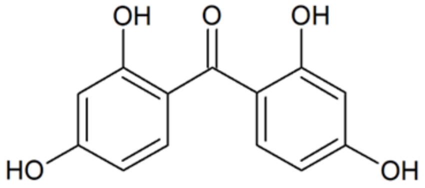

BP-2
(2,2',4,4'-tetrahydroxybenzophenone)

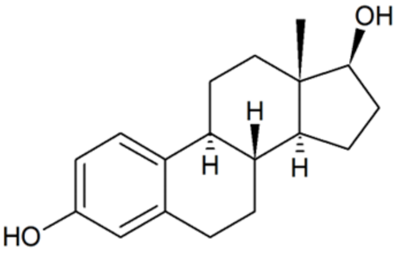

E2
(Estradiol)

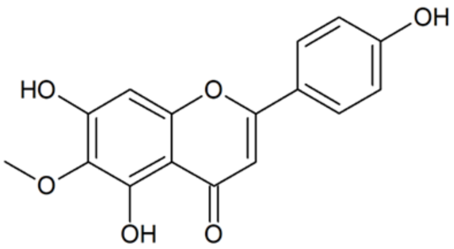

Hispidulin

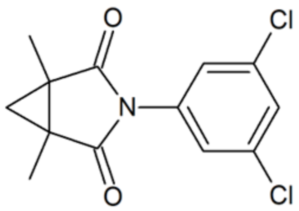

Procymidone

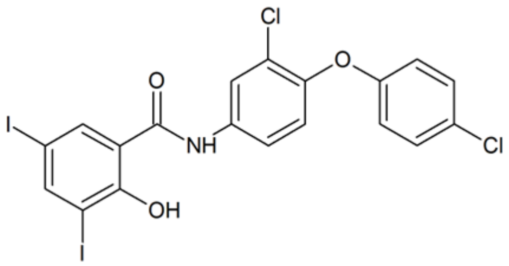

Rafoxanide

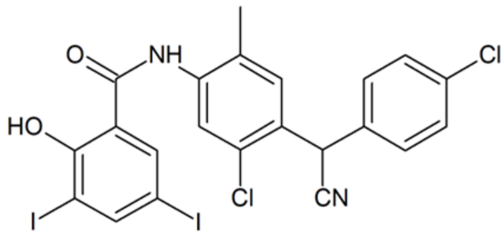

Closantel

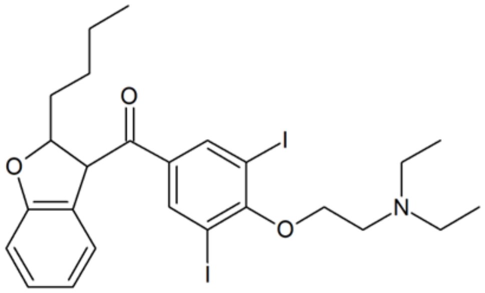

Amiodarone

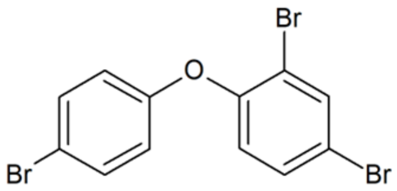

BDE28
(2,4,4'-Tribromodiphenyl ether)

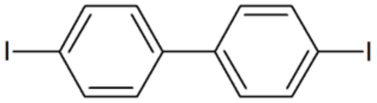

4,4’-DIDBP
(4,4’-diiodobiphenyl)

Fig. S2. MS/MS spectra of predicted biotransformants

<table>
<tr><th>Structure</th><th>Spectra</th></tr>
<tr><td>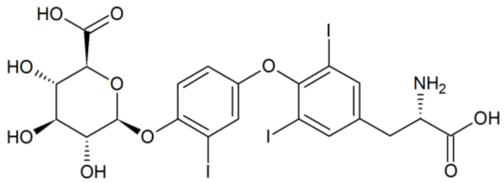
a. M1
Glucuronide Conjugation
+(C6 H8 O6)
m/z = 827.8273</td><td>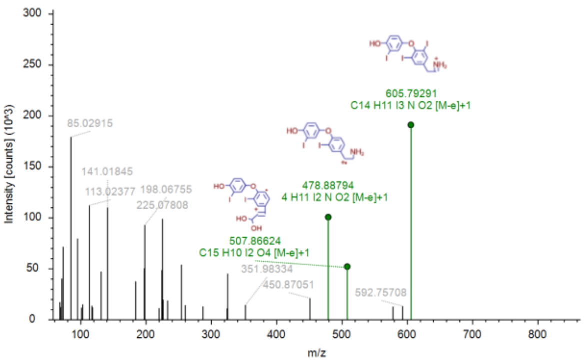
</td></tr>
<tr><td>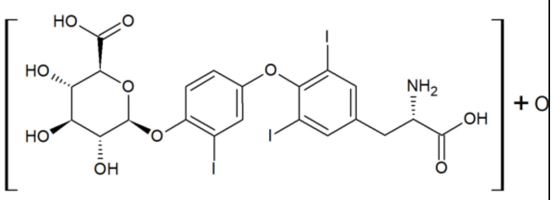
b. M2
Oxidation, Glucuronide Conjugation
+(C6 H8 O7)
m/z = 843.8254</td><td>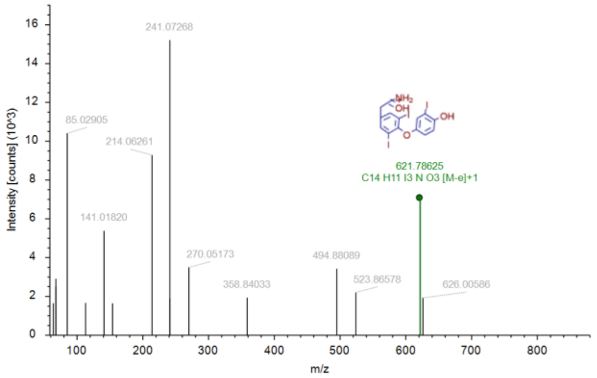
</td></tr>
<tr><td>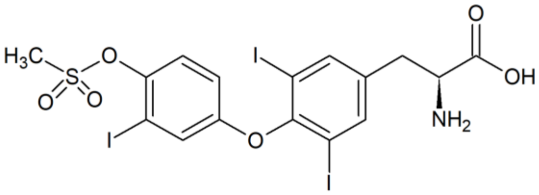
c. M4
Sulfation
+(O3 S)
m/z = 731.7549</td><td>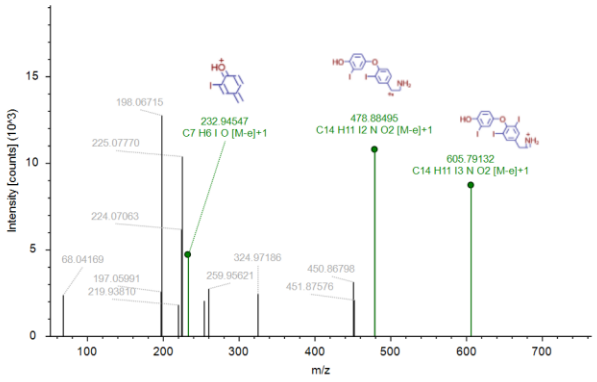
</td></tr>
</table>

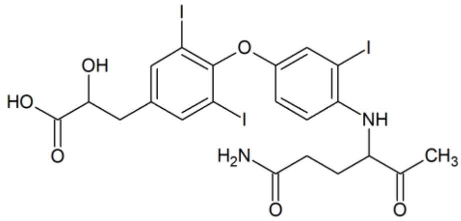

d. M5
Oxidative Deamination to Alcohol, Glutamine Conjugation
+(C5 H7 N O3)
m/z = 780.8401

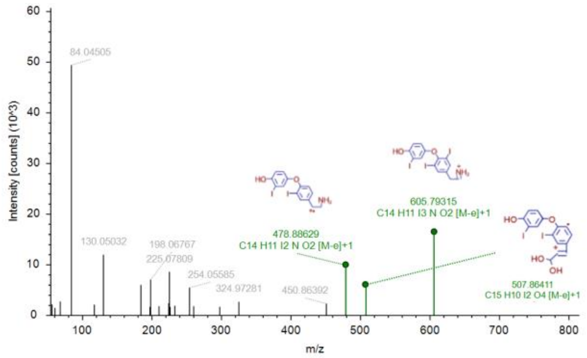

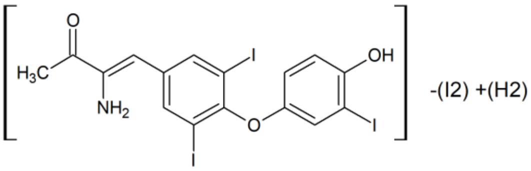

e. M22
Desaturation, Deiodination, Deiodination
-(I2)
m/z = 397.9889

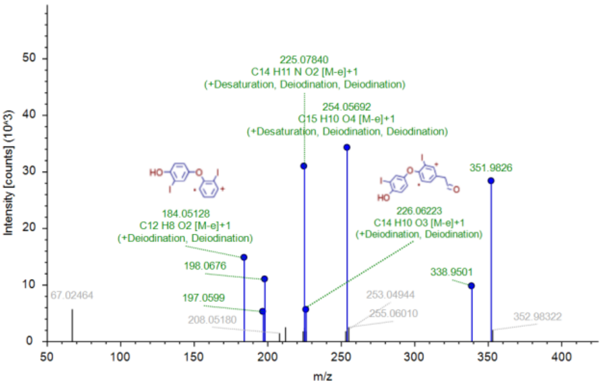

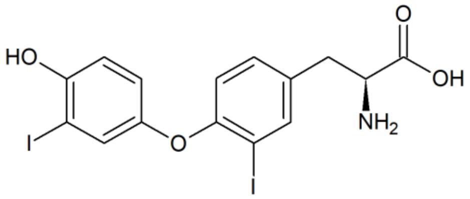

f. M27
Deiodination
-(I) +(H)
m/z = 525.9006

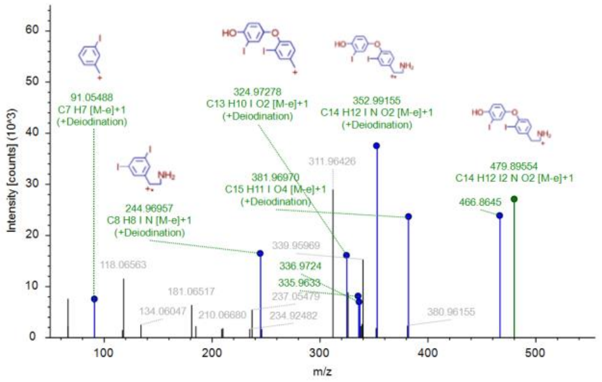

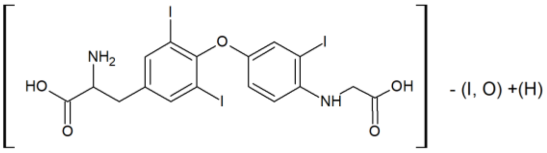

g. M28
Dehydration, Reduction, Deiodination, Glycine conjugation
-(I) +(C2 H4 N)
m/z = 566.9280

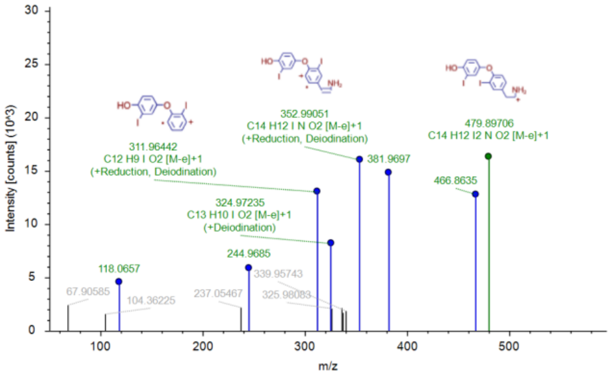

Table S1. LC-MS analysis conditions

TDCs	Final concentration of TDC	Mobile phase A	Mobile phase B	Ionization
T3	20 μM	0.1% formic acid in DW	0.1% formic acid in ACN	positive
T4	20 μM	0.1% formic acid in DW	0.1% formic acid in ACN	positive
DITPA	20 μM	0.1% formic acid in DW	0.1% formic acid in ACN	negative
GC-1	20 μM	0.1% formic acid in DW	0.1% formic acid in ACN	negative
BPA	20 μM	10 mM ammonium acetate in DW	10 mM ammonium acetate in ACN	negative
BP-2	20 μM	0.1% formic acid in DW	0.1% formic acid in ACN	negative
E2	80 μM	10 mM ammonium acetate in DW	10 mM ammonium acetate in ACN	negative
Hispidulin	20 μM	0.1% formic acid in DW	0.1% formic acid in ACN	positive
Procymidone	20 μM	0.1% formic acid in DW	0.1% formic acid in ACN	positive
Rafoxanide	20 μM	0.1% formic acid in DW	0.1% formic acid in ACN	negative
Closantel	20 μM	0.1% formic acid in DW	0.1% formic acid in ACN	negative
Amiodarone	1 μM	0.1% formic acid in DW	0.1% formic acid in ACN	positive
BDE28	20 μM	10 mM ammonium acetate in DW	10 mM ammonium acetate in ACN	negative
4,4′-DIDBP	20 μM	10 mM ammonium acetate in DW	10 mM ammonium acetate in ACN	negative

Table S2. Biotransformants of T3

FC: Fold change of activation over inactivation group.
P-value is measured by Mann-Whitney U-test.
In order to distinguish isomer of biotransformants, early-eluting feature is designated by (A) and late-eluting feature is designated by (B).

No.	Code	Composition Change	Transformations	Molecular Weight	MS/MS	RT (min.)	FISh Coverage	FC	P-value
1	M1	+(C6 H8 O6)	Glucuronide Conjugation	826.82001	MS/MS	8.3	17.65	7697.41	0.049535
2	M2	+(C6 H8 O7)	Oxidation, Glucuronide Conjugation	842.81781	MS/MS	8.108	12.5	719.56	0.049535
3	M3	+(C2 H3 N O3 S)	Desaturation, Oxidation, Taurine Conjugation	771.77448	MS/MS	8.006	0	197.79	0.049535
4	M4	+(O3 S)	Sulfation	730.74746	MS/MS	8.005	50	150.61	0.049535
5	M5	+(C5 H7 N O3)	Oxidative Deamination to Alcohol, Glutamine Conjugation	779.83297	MS/MS	10.847	33.33	59.75	0.049535
6	M6	-(I) +(C6 H9 O6)	Deiodination, Glucuronide Conjugation	700.92646	Accurate Mass	7.524	0	41.78	0.049535
7	M7	+(C3 H11 N O2 S)	Hydration, Nitro Reduction, Oxidation, Cysteine Conjugation 2	775.83847	MS/MS	11.586	0	39.70	0.049535
8	M8	+(C3 H9 N O S)	Nitro Reduction, Oxidation, Cysteine Conjugation 2_A	757.82766	MS/MS	11.586	0	32.09	0.049535
9	M9	+(C6 H4 N4)	Dehydration, Desaturation, Desaturation, Desaturation, Arginine Conjugation	782.83304	Accurate Mass	9.833	0	22.15	0.049535
10	M10	+(C2 H3 N O)	Glycine Conjugation	707.81247	Accurate Mass	12.077	0	15.74	0.049535
11	M11	+(C2 H4 O)	Reduction, Acetylation	694.81703	Accurate Mass	10.082	0	14.89	0.049535
12	M12	-(I) +(H O3 S)	Deiodination, Sulfation	604.85204	Accurate Mass	7.352	0	11.82	0.049535
13	M13	+(C H2)	Methylation	664.80547	Accurate Mass	14.051	0	11.03	0.049535
14	M14	-(N) +(H O S)	Dehydration, Nitro Reduction, Oxidative Deamination to Alcohol, Reduction, Sulfation	685.76068	Accurate Mass	0.932	0	10.18	0.049535
15	M15	-(I3 O) +(C6 H17 N4)	Nitro Reduction, Deiodination, Deiodination, Deiodination, Arginine Conjugation	399.22646	Accurate Mass	11.758	0	10.02	0.049535
16	M16	+(C6 H10 O5)	Glucoside Conjugation_B	812.84336	MS/MS	9.479	0	9.85	0.049535
17	M17	+(C6 H10 O5)	Glucoside Conjugation_A	812.84239	Accurate Mass	8.004	0	9.72	0.049535
18	M18	-(I) +(C10 H16 N3 O6 S)	Deiodination, GSH Conjugation 1	829.96203	Accurate Mass	7.234	0	9.36	0.049535
19	M19	+(C3 H9 N O S)	Nitro Reduction, Oxidation, Cysteine Conjugation 2_B	757.82768	Accurate Mass	12.046	0	6.24	0.049535
20	M20	-(N) +(C16 H33 O)	Hydration, Nitro Reduction, Oxidative Deamination to Alcohol, Palmitoyl Conjugation	878.04264	Accurate Mass	0.724	0	6.08	0.049535
21	M21	+(C O)	Desaturation, Oxidation, Methylation	678.78612	Accurate Mass	9.256	0	5.16	0.049535
22	M22	-(I2)	Desaturation, Deiodination, Deiodination_B	396.98165	Accurate Mass	9.461	0	3.77	0.049535
23	M23	-(I2) +(C2 H3 N)	Dehydration, Deiodination, Deiodination, Glycine Conjugation	438.00852	Accurate Mass	8.591	0	3.68	0.049535
24	M24	-(H I)	Desaturation, Deiodination_A	522.87878	Accurate Mass	9.31	0	3.62	0.049535
25	M25	-(H I)	Desaturation, Deiodination_B	522.8783	Accurate Mass	11.11	0	3.45	0.049535
26	M26	-(I2) +(C6 H9 N3 O)	Dehydration, Oxidative Deamination to Ketone, Deiodination, Deiodination, Arginine Conjugatior	536.05513	MS/MS	0.745	10	3.39	0.049535
27	M27	-(I) +(H)	Deiodination	524.89341	MS/MS	9.983	57.14	3.27	0.049535
28	M28	-(I) +(C2 H4 N)	Dehydration, Reduction, Deiodination, Glycine Conjugation	565.92065	MS/MS	9.983	100	3.22	0.049535
29	M29	-(I2)	Desaturation, Deiodination, Deiodination_A	396.9816	MS/MS	8.591	76.92	3.18	0.049535
30	M30	+(C16 H34 O3)	Hydration, Oxidation, Reduction, Palmitoyl Conjugation	925.0356	Accurate Mass	0.704	0	1.83	0.126630
31	M31	-(I2) +(C10 H19 N3 O5 S)	Dehydration, Reduction, Deiodination, Deiodination, GSH Conjugation 2	690.08031	MS/MS	0.873	0	1.74	0.049535
32	M32	-(I) +(C10 H18 N3 O8 S)	Oxidation, Oxidation, Deiodination, GSH Conjugation 2	863.96882	Accurate Mass	0.855	0	1.46	0.049535
33	M33	-(H I) +(C2 O2 S)	Dehydration, Desaturation, Oxidative Deamination to Ketone, Deiodination, Taurine Conjugation	610.83939	Accurate Mass	0.96	0	1.39	0.049535
34	M34	-(I2 O) +(C6 H10 N4)	Dehydration, Dehydration, Deiodination, Deiodination, Arginine Conjugation	519.07723	Accurate Mass	10.107	0	1.34	0.049535
35	M35	+(C O3)	Desaturation, Oxidation, Oxidation, Oxidation, Methylation	710.77388	Accurate Mass	0.96	0	1.26	0.275234
36	M36	-(I3 O) +(H7)	Hydration, Nitro Reduction, Deiodination, Deiodination, Deiodination	261.13661	Accurate Mass	11.863	0	1.26	0.275234
37	M37	-(I) +(H O2)	Oxidation, Oxidation, Deiodination	556.88529	Accurate Mass	0.843	0	1.15	0.126630
38	M38	+(C2 H N)	Dehydration, Glycine Conjugation	689.80139	MS/MS	12.333	28.57	1.14	0.827259
39	M39	+(C3 H6 O2 S)	Dehydration, Oxidative Deamination to Alcohol, Reduction, Cysteine Conjugation 2	756.79793	MS/MS	0.866	6.67	1.10	0.512691
40	M40	-(O2) +(C2 H4 S)	Dehydration, Nitro Reduction, Nitro Reduction, Oxidative Deamination to Ketone, Taurine Conjuc	678.8024	Accurate Mass	0.845	0	1.05	0.827259
41	M41	+(C2 H3 N)	Dehydration, Reduction, Glycine Conjugation	691.8149	MS/MS	10.64	50	1.01	0.512691
42	M42	-(N) +(C2 H O4)	Hydration, Oxidation, Oxidative Deamination to Ketone, Acetylation	725.77825	Accurate Mass	11.566	0	1.00	0.827259

Supplementary Table S3. Biotransformants of DITPA

FC: Fold change of activation over inactivation group.
P-value is measured by Mann-Whitney U-test.
In order to distinguish isomer of biotransformants, early-eluting feature is designated by (A) and late-eluting feature is designated by (B).

No.	Code	Composition Change	Transformations	Molecualr Weight	MS/MS	RT (min.)	FISh Coverage	FC	P-value
1	M1	+(C6 H8 O6)	Glucuronide Conjugation	685.91395	MS/MS	9.644	0	17447.54	0.049535
2	M2	+(C6 H8 O7)	Oxidation, Glucuronide Conjugation_B	701.90981	MS/MS	9.422	16.67	1191.81	0.049535
3	M3	-(I2) +(H2 O3 S)	Deiodination, Deiodination, Sulfation	338.04616	MS/MS	7.075	57.14	429.45	0.049535
4	M4	-(I) +(C6 H9 O6)	Deiodination, Glucuronide Conjugation	560.01812	MS/MS	8.934	0	260.92	0.049535
5	M5	+(C10 H15 N3 O5 S)	Dehydration, GSH Conjugation 2	798.95673	MS/MS	10.705	0	125.78	0.049535
6	M6	-(I2) +(C6 H14 N4)	Dehydration, Reduction, Deiodination, Deiodination, Arginine Conjugation_B	398.194	MS/MS	9.97	5.88	120.46	0.049535
7	M7	+(C6 H8 O7)	Oxidation, Glucuronide Conjugation_A	701.9098	MS/MS	9.172	20	55.75	0.049535
8	M8	-(I) +(C6 H9 N4 O2)	Desaturation, Desaturation, Oxidation, Deiodination, Arginine Conjugation	552.04941	MS/MS	0.737	6.67	35.20	0.049535
9	M9	+(O3 S)	Sulfation	589.83978	MS/MS	8.805	0	26.07	0.049535
10	M10	-(I) +(H5)	Hydration, Nitro Reduction, Oxidation, Deiodination	388.01721	MS/MS	0.75	11.11	25.25	0.049535
11	M11	-(I) +(H O3 S)	Deiodination, Sulfation	463.9429	MS/MS	8.18	33.33	18.64	0.049535
12	M12	-(I2 O) +(C6 H14 N4)	Desaturation, Nitro Reduction, Deiodination, Deiodination, Arginine Conjugation_B	382.19879	MS/MS	11.773	7.14	16.80	0.049535
13	M13	+(O)	Oxidation	525.87749	MS/MS	10.799	0	16.50	0.049535
14	M14	+(C H2 O3)	Oxidation, Oxidation, Oxidation, Methylation	571.88338	MS/MS	10.8	0	14.83	0.049535
15	M15	-(I2 O) +(C6 H14 N4)	Desaturation, Nitro Reduction, Deiodination, Deiodination, Arginine Conjugation_A	382.1991	Accurate Mass	10.946	0	13.37	0.049535
16	M16	-(I2) +(H2 O4 S)	Oxidation, Deiodination, Deiodination, Sulfation	354.041	Accurate Mass	6.988	0	12.63	0.049535
17	M17	-(I2) +(C6 H16 N4 O2)	Hydration, Deiodination, Deiodination, Arginine Conjugation_B	432.19965	MS/MS	18.569	16.67	11.22	0.049535
18	M18	+(O4 S)	Oxidation, Sulfation	605.83461	Accurate Mass	8.812	0	11.04	0.049535
19	M19	+(C10 H15 N3 O7 S)	Oxidation, GSH Conjugation 1	830.94616	MS/MS	8.836	0	9.92	0.049535
20	M20	-(I2) +(C6 H8 O6)	Desaturation, Deiodination, Deiodination, Glucuronide Conjugation	432.10606	Accurate Mass	8.015	0	9.64	0.049535
21	M21	-(I) +(C6 H9 O7)	Oxidation, Deiodination, Glucuronide Conjugation	576.01269	Accurate Mass	8.732	0	8.91	0.049535
22	M22	-(I2 O3) +(C6 H18 N4)	Nitro Reduction, Nitro Reduction, Deiodination, Deiodination, Arginine Conjugation_B	354.2401	Accurate Mass	12.739	0	8.89	0.049535
23	M23	-(I2) +(C6 H14 N4)	Dehydration, Reduction, Deiodination, Deiodination, Arginine Conjugation_A	398.19411	Accurate Mass	9.084	0	7.09	0.049535
24	M24	-(I2) +(C6 H16 N4 O2)	Hydration, Deiodination, Deiodination, Arginine Conjugation_A	432.19966	MS/MS	18.286	20	6.84	0.049535
25	M25	-(I2) +(C10 H19 N3 O3 S)	Dehydration, Nitro Reduction, Deiodination, Deiodination, GSH Conjugation 2_B	517.18965	Accurate Mass	18.828	0	5.96	0.049535
26	M26	-(I2 O) +(C6 H10 N4)	Dehydration, Dehydration, Deiodination, Deiodination, Arginine Conjugation_B	378.17112	MS/MS	18.881	12.5	5.74	0.049535
27	M27	-(I2) +(C10 H17 N3 O3 S)	Dehydration, Nitro Reduction, Deiodination, Deiodination, GSH Conjugation 1_A	515.17364	MS/MS	18.282	0	4.74	0.049535
28	M28	-(I) +(C2 H7)	Hydration, Nitro Reduction, Deiodination, Acetylation	414.03151	Accurate Mass	20.177	0	4.28	0.512691
29	M29	-(I2) +(C6 H10 O5)	Desaturation, Deiodination, Deiodination, Glucoside Conjugation	418.1266	Accurate Mass	7.102	0	3.68	0.049535
30	M30	-(I2) +(H2 O2 S)	Dehydration, Reduction, Deiodination, Deiodination, Sulfation	322.05094	MS/MS	6.613	22.22	2.76	0.049535
31	M31	-(I2) +(C18 H34)	Dehydration, Deiodination, Deiodination, Stearyl Conjugation	506.34099	Accurate Mass	21.413	0	2.18	0.12663
32	M32	-(I2 O) +(C6 H10 N4)	Dehydration, Dehydration, Deiodination, Deiodination, Arginine Conjugation_A	378.16874	MS/MS	11.929	0	1.62	0.512691
33	M33	+(C6 O4)	Dehydration, Dehydration, Desaturation, Desaturation, Glucuronide Conjugation	645.85877	Accurate Mass	24.336	0	1.48	0.512691
34	M34	-(I O) +(C2 H3)	Desaturation, Nitro Reduction, Deiodination, Acetylation	394.00662	Accurate Mass	10.666	0	1.43	0.049535
35	M35	+(C2 H5 N O4)	Hydration, Oxidation, Oxidation, Glycine Conjugation	616.90608	Accurate Mass	0.843	0	1.39	0.12663
36	M36	-(I2 O3) +(C6 H18 N4)	Nitro Reduction, Nitro Reduction, Deiodination, Deiodination, Arginine Conjugation_A	354.24064	MS/MS	10.16	14.29	1.39	0.049535
37	M37	-(H4 O3)	Dehydration, Desaturation, Desaturation, Nitro Reduction	457.86883	Accurate Mass	24.377	0	1.31	0.12663
38	M38	-(I2) +(C2 H4 O3)	Oxidation, Oxidation, Deiodination, Deiodination, Acetylation	332.09086	Accurate Mass	7.558	0	1.31	0.12663
39	M39	-(I O4) +(H5)	Nitro Reduction, Nitro Reduction, Deiodination	324.03582	MS/MS	23.981	0	1.27	0.275234
40	M40	+(H2 O6 S)	Hydration, Oxidation, Oxidation, Sulfation	639.84034	MS/MS	11.801	0	1.26	0.512691
41	M41	-(I) +(C10 H16 N3 O6 S)	Deiodination, GSH Conjugation 1	689.05458	MS/MS	8.562	0	1.14	0.827259
42	M42	-(I O) +(C H)	Dehydration, Deiodination, Methylation	379.99174	MS/MS	10.271	10	1.13	0.827259
43	M43	-(I2) +(C H4 O2)	Oxidation, Oxidation, Deiodination, Deiodination, Methylation	304.09456	Accurate Mass	9.692	0	1.10	0.512691

Table S4. Biotransformants of GC-1

FC: Fold change of activation over inactivation group.
P-value is measured by Mann-Whitney U-test.
In order to distinguish isomer of biotransformants, early-eluting feature is designated by (A) and late-eluting feature is designated by (B).

No.	Code	Composition Change	Transformations	Molecular Weight	MS/MS	RT (min.)	FISh Coverage	FC	P-value
1	M1	+(C6 H8 O6)	Glucuronide Conjugation	504.19933	MS/MS	10.177	17.65	303.03	0.049535
2	M2	+(C4 H6 O4)	Glucuronide Conjugation, Decarboxylation	446.19342	MS/MS	10.08	11.11	26.94	0.049535
3	M3	+(O2)	Oxidation, Oxidation	360.15657	MS/MS	10.771	12.5	4.45	0.049535
4	M4	+(C10 H15 N3 O6 S)	GSH Conjugation 1	633.23571	Accurate Mass	8.529	0	2.76	0.049535
5	M5	-(H4 O) +(C2)	Dehydration, Dehydration, Desaturation, Acetylation	332.14013	Accurate Mass	6.777	0	2.63	0.049535
6	M6	+(C6 H14 O6)	Hydration, Reduction, Glucoside Conjugation_A	510.24587	Accurate Mass	7.738	0	1.79	0.049535
7	M7	+(O)	Oxidation	344.16179	MS/MS	10.681	12.5	1.71	0.275234
8	M8	+(C6 H14 O6)	Hydration, Reduction, Glucoside Conjugation_B	510.24592	MS/MS	10.592	0	1.58	0.049535
9	M9	-(O2) +(C2 H8)	Hydration, Nitro Reduction, Nitro Reduction, Acetylation	328.23973	MS/MS	18.298	0	1.41	0.049535
10	M10	+(H2 O6 S)	Hydration, Oxidation, Oxidation, Sulfation	458.12492	MS/MS	13.005	28.57	1.11	0.512691
11	M11	+(C2 H3 N O)	Glycine Conjugation	385.18846	MS/MS	9.856	0	1.06	0.049535
12	M12	+(C6 H14 N4 O)	Reduction, Arginine Conjugation	486.2858	MS/MS	14.329	16.67	1.05	0.049535
